# Neoadjuvant chemoradiotherapy plus surgery versus active surveillance for oesophageal cancer: a stepped-wedge cluster randomised trial

**DOI:** 10.1186/s12885-018-4034-1

**Published:** 2018-02-06

**Authors:** Bo Jan Noordman, Bas P. L. Wijnhoven, Sjoerd M. Lagarde, Jurjen J. Boonstra, Peter Paul L. O. Coene, Jan Willem T. Dekker, Michael Doukas, Ate van der Gaast, Joos Heisterkamp, Ewout A. Kouwenhoven, Grard A. P. Nieuwenhuijzen, Jean-Pierre E. N. Pierie, Camiel Rosman, Johanna W. van Sandick, Maurice J. C. van der Sangen, Meindert N. Sosef, Manon C. W. Spaander, Roelf Valkema, Edwin S. van der Zaag, Ewout W. Steyerberg, J. Jan B. van Lanschot, on behalf of the SANO-study group, B. J. Noordman, J. J. B. van Lanschot, S. M. Lagarde, B. P. L. Wijnhoven, K. Biermann, A. van der Gaast, E. Ista, N. C. Krak, J. J. M. E. Nuyttens, S. Polinder, M. C. W. Spaander, E. W. Steyerberg, R. Valkema, A. Agool, J. van Baarlen, E. M. Hendriksen, R. Hoekstra, E. A. Kouwenhoven, A. van der Linde, A. Bartels-Rutten, J. van Dieren, J. van Sandick, P. Snaebjornsson, E. Vegt, F. E. M. Voncken, H. Doornewaard, G. W. Erkelens, G. S. Madretsma, E. S. van der Zaag, M. R. J. ten Broek, R. J. Dallinga, J. W. T. Dekker, V. O. Dezentjé, R. R. de Krijger, K. J. Neelis, R. Quispel, G. J. Creemers, G. A. P. Nieuwenhuijzen, M. C. van der Sangen, E. J. Schoon, D. N. J. Wyndaele, J. Buijsen, R. G. Riedl, W. M. J. Schreurs, M. N. Sosef, L. E. Oostenbrug, F. A. R. M. Warmerdam, J. J. Boonstra, M. Slingerland, W. O. de Steur, I. M. Lips, W. E. Fiets, K. van der Linde, J. Nieken, V. Oppedijk, J. P. E. N. Pierie, R. Wolf, P. P. L. O. Coene, I. Al Butaihi, M. Kliffen, E. M. M. Kuiper, E. F. Courrech Staal, M. J. R. Janssen, M. H. Liedenbaum, C. van der Post, S. A. Radema, C. Rosman, H. Rütten, P. D. Siersema, L. V. Beerepoot, W. L. Hazen, J. Heisterkamp, J. C. van Oord, T. Rozema, I. A. C. Vermeltfoort, A. A. M. van der Wurff

**Affiliations:** 1 Department of Surgery, Erasmus MC – University Medical Centre, Suite Z-839, P.O. Box 2040 3000, CA Rotterdam, The Netherlands; 2 Department of Gastroenterology, Leiden University Medical Centre, Leiden, the Netherlands; 3 Department of Surgery, Maasstad Hospital, Rotterdam, the Netherlands; 4 Department of Surgery, Reinier de Graaf Group, Delft, the Netherlands; 5 Department of Pathology, Erasmus MC – University Medical Centre, Rotterdam, the Netherlands; 6 Department of Medical Oncology, Erasmus MC – University Medical Centre, Rotterdam, the Netherlands; 7 Department of Surgery, Elisabeth Tweesteden Hospital, Tilburg, the Netherlands; 8 Department of Surgery, Zorggroep Twente, Almelo, the Netherlands; 9 Department of Surgery, Catharina Hospital, Eindhoven, the Netherlands; 10 Department of Surgery, Medical Centre Leeuwarden, Leeuwarden, the Netherlands; 11 Department of Surgery, Radboud University Medical Centre, Nijmegen, the Netherlands; 12 Department of Surgery, The Netherlands Cancer Institute - Antoni van Leeuwenhoek Hospital, Amsterdam, the Netherlands; 13 Department of Radiation Oncology, Catharina Hospital, Eindhoven, the Netherlands; 14 Department of Surgery, Zuyderland Medical Centre, Heerlen, the Netherlands; 15 Department of Gastroenterology, Erasmus MC – University Medical Centre, Rotterdam, the Netherlands; 16 Department of Radiology and Nuclear Medicine, Erasmus MC – University Medical Centre, Rotterdam, the Netherlands; 17 Department of Surgery, Gelre Hospital, Apeldoorn, the Netherlands; 18 Department of Medical Statistics and Bioinformatics, Leiden University Medical Centre, formerly department of Public Health, Erasmus MC – University Medical Centre Rotterdam, Rotterdam, the Netherlands

**Keywords:** Oesophageal cancer, Neoadjuvant chemoradiotherapy, Active surveillance, Standard oesophagectomy

## Abstract

**Background:**

Neoadjuvant chemoradiotherapy (nCRT) plus surgery is a standard treatment for locally advanced oesophageal cancer. With this treatment, 29% of patients have a pathologically complete response in the resection specimen. This provides the rationale for investigating an active surveillance approach. The aim of this study is to assess the (cost-)effectiveness of active surveillance vs. standard oesophagectomy after nCRT for oesophageal cancer.

**Methods:**

This is a phase-III multi-centre, stepped-wedge cluster randomised controlled trial. A total of 300 patients with clinically complete response (cCR, i.e. no local or disseminated disease proven by histology) after nCRT will be randomised to show non-inferiority of active surveillance to standard oesophagectomy (non-inferiority margin 15%, intra-correlation coefficient 0.02, power 80%, 2-sided α 0.05, 12% drop-out). Patients will undergo a first clinical response evaluation (CRE-I) 4–6 weeks after nCRT, consisting of endoscopy with bite-on-bite biopsies of the primary tumour site and other suspected lesions. Clinically complete responders will undergo a second CRE (CRE-II), 6–8 weeks after CRE-I. CRE-II will include 18F–FDG-PET-CT, followed by endoscopy with bite-on-bite biopsies and ultra-endosonography plus fine needle aspiration of suspected lymph nodes and/or PET- positive lesions. Patients with cCR at CRE-II will be assigned to oesophagectomy (first phase) or active surveillance (second phase of the study). The duration of the first phase is determined randomly over the 12 centres, i.e., stepped-wedge cluster design. Patients in the active surveillance arm will undergo diagnostic evaluations similar to CRE-II at 6/9/12/16/20/24/30/36/48 and 60 months after nCRT. In this arm, oesophagectomy will be offered only to patients in whom locoregional regrowth is highly suspected or proven, without distant dissemination. The main study parameter is overall survival; secondary endpoints include percentage of patients who do not undergo surgery, quality of life, clinical irresectability (cT4b) rate, radical resection rate, postoperative complications, progression-free survival, distant dissemination rate, and cost-effectiveness. We hypothesise that active surveillance leads to non-inferior survival, improved quality of life and a reduction in costs, compared to standard oesophagectomy.

**Discussion:**

If active surveillance and surgery as needed after nCRT leads to non-inferior survival compared to standard oesophagectomy, this organ-sparing approach can be implemented as a standard of care.

## Background

Oesophageal cancer is an aggressive disease with poor outcomes after primary surgery [1]. Since the introduction of neoadjuvant chemo (radio) therapy, survival rates have improved substantially [2]. The randomised ChemoRadiotherapy for Oesophageal cancer followed by Surgery Study (CROSS) showed an absolute 5-year overall survival benefit of 14% after neoadjuvant chemoradiotherapy (nCRT) plus surgery, compared to surgery alone [3, 4]. Moreover, after nCRT according to CROSS, 29% of all patients (49% for squamous cell carcinoma [SCC] and 23% for adenocarcinoma [AC]) had a pathologically complete response (pCR) in the resection specimen [3]. This high pCR-rate provides the rationale to explore an organ-sparing active surveillance approach after nCRT since, intuitively, an oesophagectomy in patients with no viable residual tumour does not improve oncological outcome. In this organ-sparing treatment strategy, patients will undergo frequent diagnostic evaluations after nCRT. An oesophagectomy will be performed only in patients with a proven or high suspicion of locoregional regrowth, in the absence of distant metastases. This treatment strategy would have great advantages, especially given the perioperative morbidity and mortality, and the lasting impact on patients’ health-related quality of life (HRQOL) that is associated with oesophagectomy [3, 5–9]. An active surveillance approach would not only benefit patients who are cured by nCRT alone, but also patients with undetectable distant metastases (i.e. micrometastases) after completion of nCRT. Currently, patients with occult distant metastases undergo standard oesophagectomy. This theoretically is of no benefit, because distant metastases, which are the main determinants of long-term survival, are below the detection limit at the first clinical evaluation after nCRT. During active surveillance, these occult metastases might become clinically manifest, which will prevent patients from a non-beneficial oesophagectomy.

At present, active surveillance is applied in selected patients who refuse oesophagectomy or who are finally considered unfit for surgery after nCRT [10–13]. Explorative retrospective studies in these patients show promising results, with comparable long-term survival for active surveillance vs immediate standard surgery and comparable outcomes of postponed oesophagectomy in patients who develop a locoregional regrowth in the absence of distant metastases [10–13].

In the recently completed diagnostic preSANO-trial, endoscopy with bite-on-bite biopsies and ultra-endosonography with fine needle aspiration (FNA) of suspected lymph nodes for detection of locoregional residual disease, combined with 18F–FDG PET-CT for detection of interval metastases was adequate for clinical response evaluation after nCRT for oesophageal cancer. Using two rounds of clinical response evaluations (CREs), sensitivity and specificity for differentiation between tumour regression grade (TRG) 3–4 (i.e. > 10% vital cells) and TRG 1 (i.e. no vital cells) residual tumour using endoscopy with bite-on-bite biopsies and FNA were 90% and 72%, respectively. 18F–FDG PET-CT after nCRT detected interval metastases in 10% of patients [14].

The results of the preSANO-trial in combination with results in the literature on the clinical outcome of active surveillance justify a phase-III trial, comparing active surveillance with standard surgery in patients with a clinically complete response after nCRT.

### Objective

The aim of this study is to assess the (cost-)effectiveness (including non-financial costs and survival) of active surveillance after nCRT - as compared to standard surgery - for patients with SCC or AC of the oesophagus or oesophagogastric junction.

## Methods

### Study design

**Fig. 1:**
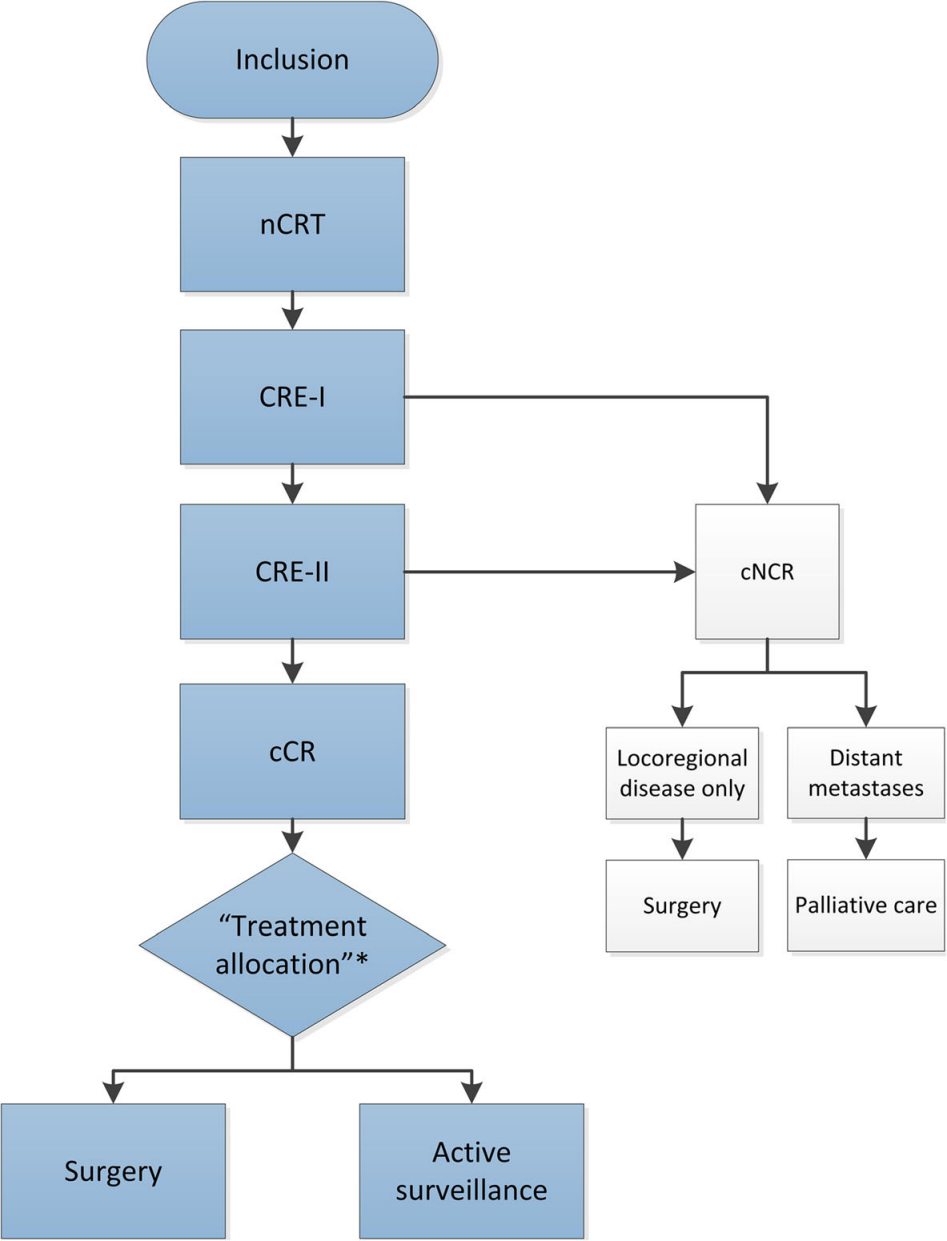
Study algorithm. nCRT: neoadjuvant chemoradiotherapy; CRE: clinical response evaluation; cNCR: clinically non-complete response; cCR: clinically complete response. *At this point the patient will be allocated to one of the two treatment arms, dependent on the institution in which the actual treatment takes place. Randomisation will be performed at the institutional level (see §3.1 and §8.2). Patients will know their allocated treatment at the moment of inclusion

**Fig. 2:**
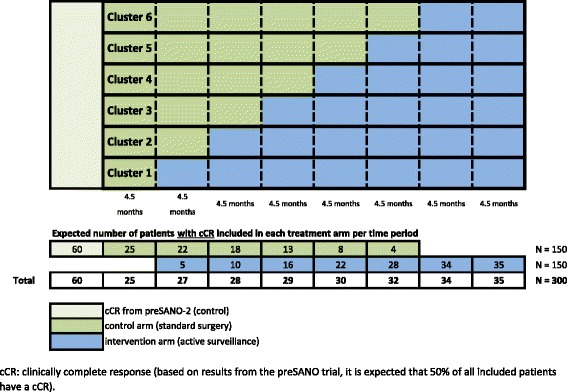
Stepped-wedge cluster design with addition of preSANO cCR-patients and sequential cross-over of 6 clusters comprising 2 centres every 4.5 months

The SANO-trial is a phase III multi-centre, stepped-wedge, cluster randomised controlled non-inferiority trial. This design involves random sequential switch of clusters of participating institutions from the control arm (standard surgery) to the interventional arm (active surveillance). Randomisation is performed at the institutional level, instead of the individual level (Figs. 1 and 2) [15]. Twelve high-volume centres in the Netherlands are participating in this study (Erasmus Medical Centre, Rotterdam; Catharina Cancer Institute, Eindhoven; Zuyderland Medical Centre, Heerlen; Radboud University Medical Centre, Nijmegen; Elisabeth Tweesteden Hospital, Tilburg; Gelre Hospital, Apeldoorn; Leiden University Medical Centre, Leiden; Maasstad Hospital, Rotterdam; Zorggroep Twente, Almelo; Netherlands Cancer Institute, Amsterdam; Reinier de Graaf Group, Delft; Medical Centre Leeuwarden). Based on these 12 participating centres, 6 clusters with comparable estimated inclusion rates will be formed, each cluster comprising 2 participating centres. Based on the expected inclusion period of 36 months and the inclusion of 60 clinically complete responders from the preSANO trial (see below; Statistical Analysis; Sample Size Calculation), every 4.5 months one cluster will switch from the control arm to the interventional arm. Clusters will be determined by randomisation, but always consist of a centre with high expected total inclusion (≥45) and a centre with a lower (< 30) expected total inclusion.

During the first 4.5 months of the trial, all centres will provide standard immediate surgery and will gain experience in the performance of clinical response (and surveillance) evaluations. After 4.5 months, a cluster of 2 centres (Erasmus MC and Zuyderland Medical Centre) with extensive experience in CREs and a large number of patients included in the preSANO-trial, will start to provide the novel strategy (active surveillance). After the next 4.5 months, another cluster of 2 participating centres will be randomly assigned by the sponsor using a computer-generated number sequence to begin with active surveillance. This procedure will be repeated after 4.5 months until all clusters have crossed over into the active surveillance arm. The final phase of the trial, with all sites including patients in the active surveillance arm, finishes approximately 9 months after the last cluster of two sites have switched from the control arm to the interventional arm (Fig. 2).

Patients who prefer the treatment that is not offered as study treatment in that particular centre at that time (e.g. active surveillance in a centre that has not yet crossed over into the active surveillance group) cannot be included in the trial. These patients will still be treated in the same centre, but outside the trial.

Expected numbers of patients included in both study arms during the different time periods and predefined clusters with comparable expected numbers of inclusions are shown in Fig. 2. Inclusion rate will be closely monitored during the trial, and time periods will be adjusted if the number of included patients differ substantially from the expectations.

### Study population

Operable patients with locally advanced resectable SCC or AC of the oesophagus or oesophagogastric junction who are planned to undergo nCRT according to CROSS followed by surgical resection are eligible for inclusion [3]. Patients with language difficulties, dementia or altered mental status prohibiting the understanding and giving of informed consent and patients with non-FDG-avid tumours at baseline will be excluded from participation in this study. Patients will have conventional pre-treatment work-up (including F18-FDG PET-CT to assess the avidity of the primary tumour).

### Study algorithm (Table 1, Fig. 1, Fig. 3)

All included patients will undergo nCRT according to CROSS (Carboplatin AUC 2 mg/mL per min, Paclitaxel 50 mg/m2 of body-surface area and 41.4 Gy of concurrent radiotherapy in 23 fractions) [3]. Patients will be re-staged after nCRT during CREs to select those who may benefit from active surveillance. CREs categorise patients as clinically complete responders or clinically incomplete responders. Only patients in whom no locoregional or disseminated disease is proven (cCR) during CREs, will be included in the comparative part of this trial.

**Table 1:** Study algorithm

	Pretreatment	CRE-I (4–6 weeks after nCRT)	CRE-II (10–14 weeks after nCRT)	Standard surgery arm (6, 9, 12, 16, 20, 24, 30, 36, 48 and 60 months after nCRT)	Active surveillance arm (6, 9, 12, 16, 20, 24, 30, 36, 48 and 60 months after nCRT)
Informed consent	X				
Inclusion			X		
Treatment allocation^h^			X		
ECOG performance status	X	X	X	X	X
Endoscopy with bite-on-bite biopsies	X	X	X		X
Radial EUS	X		X		X
Linear EUS with FNA of suspected lymph nodes	X		X		X
18F–FDG PET-CT (whole-body)	X	X^a^	X^b^	X^c^	X^b^
Quality of Life (EQ-5D, QLQ-C30, QLC-OG25 en Cancer Worry Scale)	X		X	X^d^	X^d^
Oesophagectomy		X^e^	X^f^	All	At indication^g^

^a^18F–FDG PET-CT: during CRE-I, after OGD, only for clinically non-complete responders, to exclude disseminated disease

^b^18F–FDG PET-CT: during CRE-II and active surveillance, prior to OGD and EUS, for all patients (all were clinically complete responders during CRE-I) to guide endoscopists in taken biopsies / FNA during OGD and EUS and to exclude disseminated disease

^c^ PET-CT in the standard surgery arm will be performed at 12 and 24 months after nCRT only, to exclude disseminated disaese

^d^Quality of life will be assessed during the first 2 years only

^e^ Only for patients with locoregional disease

^f^ After CRE-II: Only for patients with cCR who are allocated to surgery

^g^ Only for patients in whom a locoregional regrowth is highly suspected or proven, without any signs of distant dissemination

CRE: clinical response evaluation; nCRT: neoadjuvant chemoradiotherapy; ECOG: Eastern Cooperative Oncology Group EUS: endo-ultrasonograpy; FNA: fine needle aspiraton. ^h^At this point the patient will be allocated to one of the two treatment arms, dependent on the institution. Randomisation has already been performed at the institutional level and will be known to the patient at the moment of inclusion

**Fig. 3:**
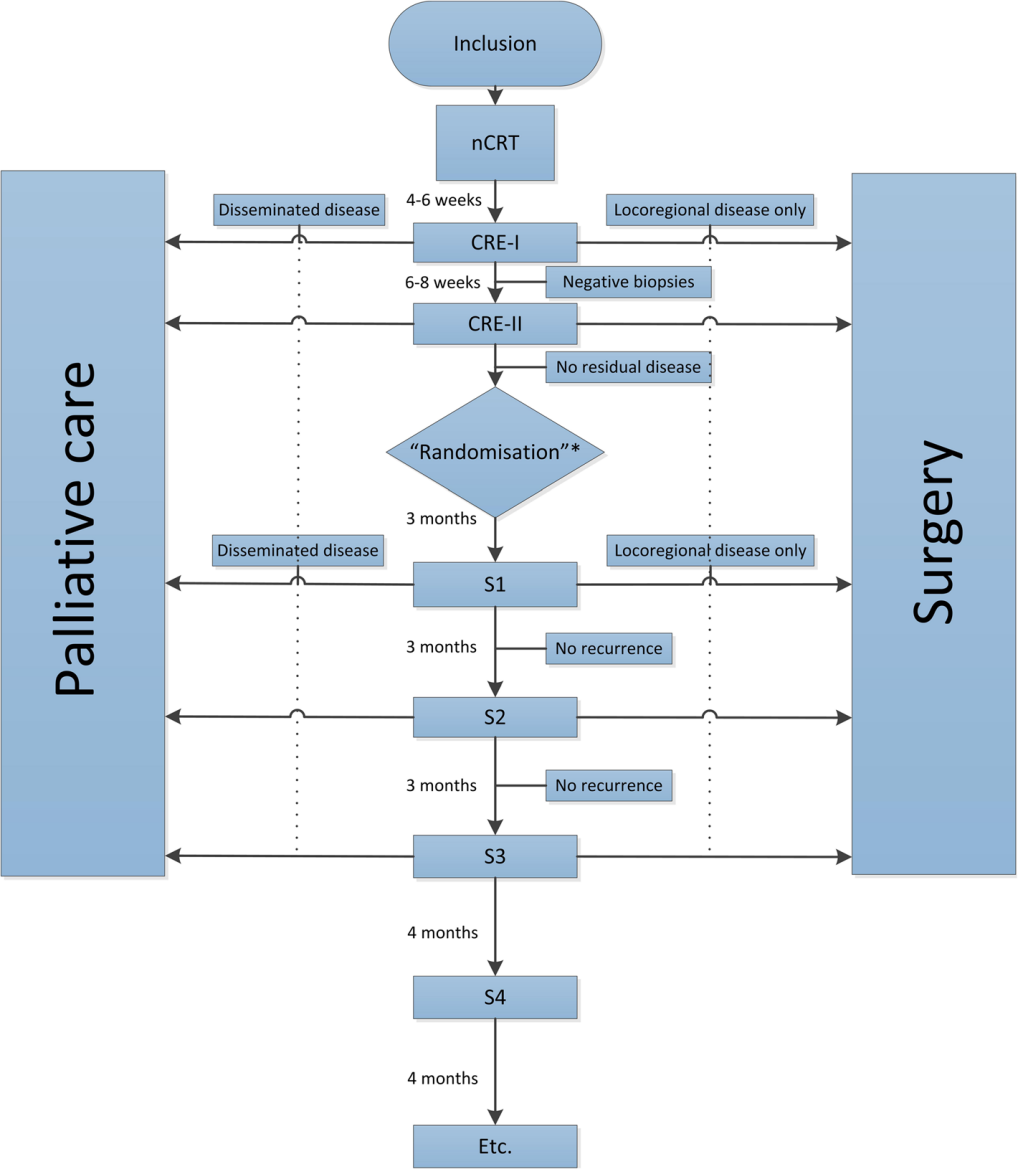
Expected distribution of patients. nCRT: neoadjuvant chemoradiotherapy; CRE: clinical response evaluation; S1: first surveillance evaluation; S2: second surveillance evaluation etc. Treatment allocation*: randomisation will be performed at institutional level and will be known already at the moment of inclusion; immediate surgery arm of randomisation not shown

#### CREs

Approximately 4–6 weeks after completion of nCRT all included patients will undergo a first clinical response evaluation (CRE-I) including oesophagogastroduodenoscopy (OGD) with at least 8 (random) biopsies, including at least 4 bite-on-bite biopsies of the primary tumour site and of any other suspected lesions. Patients with (cyto) histological evidence of locoregional residual disease during CRE-I will be offered a subsequent 18F–FDG PET-CT to exclude disseminated disease and will be offered immediate surgery (i.e. 6–8 weeks after completion of nCRT). Patients who are found to be cCR will undergo a second CRE (CRE-II) 6–8 weeks after CRE-I (i.e. 10–14 weeks after completion of nCRT). CRE-II will include an 18F–FDG PET-CT, followed by OGD with bite-on-bite biopsies of the primary tumour site and any other suspected lesions, radial EUS and in case of PET-positive lesions and/or suspected lymph nodes, even if these lymph nodes are located directly adjacent to the primary tumour site, linear EUS with FNA. The 18F–FDG PET-CT during CRE-II must be available to guide the endoscopist in taking biopsies and FNA during OGD and EUS. Patients with (cyto)histological evidence of locoregional residual disease or highly suspected locoregional residual disease on 18F–FDG PET-CT, and without distant metastases during CRE-II will undergo surgery immediately after CRE-II (i.e. 10–14 weeks after completion of nCRT). Patients with distant metastases will be referred for palliative care.

Patients without (cyto)histological evidence of residual disease during CRE-II (cCR), in the absence of distant metastases, will be assigned to active surveillance (experimental arm) or standard surgery (control arm), according to the randomisation at the institutional level.

#### Active surveillance

Patients in the active surveillance arm will undergo active surveillance by 18F–FDG PET-CT, OGD with at least 8 biopsies, including at least 4 bite-on-bite biopsies and EUS plus FNA of all suspected lymph nodes at 6, 9, 12, 16, 20, 24, 30, 36, 48 and 60 months after completion of nCRT or when symptoms or results of any diagnostic test require shorter assessment intervals. Patients with (cyto)histological evidence of disseminated disease during active surveillance will be referred for palliative care (Fig. 3).

#### Surgery

All patients in the control arm without distant metastases will be offered oesophagectomy after CRE-II, whereas patients in the active surveillance arm will be offered surgery only when locoregional regrowth is highly suspected or proven, also without any signs of distant dissemination (Fig. 3).

A transthoracic oesophagectomy or a transhiatal oesophagectomy will be performed, depending on both patient characteristics and local expertise and preference. Open, hybrid and completely minimally invasive techniques are allowed. At least 15 lymph nodes should be harvested in every patient. An en-bloc resection of the primary tumour and the regional lymph nodes should be carried out including a standard dissection of the lymph nodes around the coeliac axis (separately collected for nodes along the left gastric, common hepatic and splenic artery). In the chest, at least the right paratracheal, subcarinal and para-oesophageal lymph nodes should be harvested.

#### Pathology

All CRE- and surveillance biopsies will be assessed by expert GI pathologists. Initially, all biopsies will be analysed based on the regular HE-slides (which contains two or three levels). If analysis at these levels reveals obvious vital tumour, the biopsy will be classified (diagnosed) as positive. If the assessment of this HE-slide is negative for malignancy (no malignancy), deeper sections will be performed (two or three additional levels, depending on the amount of tissue on the paraffin block). In case of doubt regarding the presence of tumour (cells) after analysis of a biopsy at the aforementioned additional levels, extra dPAS and (pan)keratin staining will be performed. In case of an originally diagnosed signet-ring cell carcinoma or a poorly cohesive carcinoma with mucin production, analysis at three additional (deeper) levels and dPAS and keratin staining will be performed consistently.

Only the CRE- and surveillance biopsies with uncertain outcome will be revised at the Department of Pathology of the Erasmus MC following the same strategy.

The resection specimens will be assessed using the 7th edition of the UICC TNM cancer staging. Microscopically radical resection (R0) will be defined as a tumour-free resection margin (margin > 1 mm not required). Also, prepTNM staging will be estimated as described earlier [16]. Tumour regression grade (TRG) will be determined according to the modified Mandard classification (TRG 1 to 4) [14].

#### Centralised multidisciplinary tumour board

During CRE-I and CRE-II, positive (cyto)histology is preferably available when offering a patient surgical resection. However, during active surveillance we do allow a centralised multidisciplinary tumour board (MTB, Erasmus MC) to recommend surgical resection in selected patients who have a high clinical / diagnostic suspicion of tumour regrowth, despite repeatedly negative (cyto)histology. This centralised MTB will monitor and decide on all such suspected patients from all participating centres. The reason for offering surgical resection in patients with a (strong) clinical suspicion of regrowth, but without positive (cyto)histology is to minimise the risk that a difficulty in confirming regrowth by histology causes a delay that will permit a tumour regrowth to expand into an irresectable stage. If for instance the intensity of a hotspot on 18F–FDG PET-CT substantially increases over time during surveillance but positive (cyto)histology cannot be obtained, the MTB can decide to recommend surgery.

### Follow-up

Follow-up visits of patients in both study arms will occur at 6, 9, 12, 16, 20, 24, 30, 36, 48 and 60 months after completion of nCRT. Additional visits will be scheduled if complaints will arise before the next visit. In cases of suspected recurrence, thoraco-abdominal CT, PET-CT and/or upper gastrointestinal endoscopy will be performed. In order to accurately compare distant dissemination rates between both treatment arms, 18F–FDG PET-CT scan will be performed in all patients in the standard surgery arm after 1 and 2 years of follow-up, after which most (> 80% and > 90%, resp.) distant metastases will likely have been detected [17]. If a patient in the active surveillance will undergo postponed oesophagectomy due to a locoregional regrowth without distant metastases, follow-up will be performed according to the Dutch Guideline for oesophageal cancer [18].

### Study parameters/endpoints

The main study parameter in this study is overall survival of patients with cCR at CRE-II (i.e. 10*–*14 weeks after completion of nCRT). Secondary study parameters include:

- The percentage of patients in the active surveillance arm who do not undergo surgery (i.e. patients who are cured by nCRT or who have occult distant metastases during initial staging, which become manifest during active surveillance);
- HRQOL as measured with EQ-5D [19], QLQ-C30 [20], QLC-OG25 [21] and Cancer Worry Scale [22] questionnaires;
- Clinical irresectability (cT4b) rate; R_0_-resection rate defined as percentage of patients within the entire randomised population who undergo resection, defined as a tumour-free resection margin;
- Postoperative morbidity/complications for all randomised patients with cCR who undergo resection, as defined by the Esophageal Complications Consensus Group [23];
- Postoperative mortality for all patients with cCR who undergo resection, defined as 90 day- and/or in-hospital mortality;
- Progression-free survival, defined as the interval between randomisation and the earliest occurrence of disease progression resulting in primary (or peroperative) irresectability of disease, locoregional regrowth (after completion of therapy);
- Distant dissemination rate;
- Cost-effectiveness.

### Safety and stopping rules

Delaying surgical resection in patients in the active surveillance arm should neither lead to a significant reduction in tumour resectability and radical resection rate, nor to a significant increase in postoperative mortality and distant dissemination rate. Therefore, the following parameters are closely monitored;

- Proportion of all patients in the active surveillance arm that present with an irresectable or incurable (T4b or R2) regrowth, in the absence of distant metastases;
- Proportion of all patients in the active surveillance arm that undergo a microscopically non-radical (R1) resection;
- Postoperative morbidity; postoperative in-hospital mortality in all patients in the active surveillance arm, proportion of all patients in the active surveillance arm with hospital stay > 60 days or who develop postoperative trachea-neo-oesophageal fistula;
- Proportion of all patients in the active surveillance arm that develop distant dissemination after 1 and 2 years of follow-up.

If outcomes of one or more of these parameters in the active surveillance arm significantly exceed the outcomes in the standard surgery arm or in the Dutch Upper-GI Cancer Audit (DUCA) data 2016, all participating centres will be notified immediately and further inclusion will be stopped [24]. Patients who have been already included will be informed and offered the possibility of immediate (high-priority) surgical resection, even in the absence of suspicion of regrowth. Continuation of active surveillance will also still be offered.

### Statistical analysis

#### Sample size calculation

In the present phase-III study, we plan to randomise at institutional level 300 patients with cCR during CRE-II between active surveillance and standard surgical resection. Simulation of trial outcomes with expected equal 3-year overall survival rates of 67% in both trial arms and an intra-correlation coefficient of 0.02 to account for between-institution variation (inter-quartile range for 3-year overall survival rates of 63%–71%) indicates a total sample size of 264 patients to show non-inferiority of surveillance to standard surgery with 80% power [25]. Non-inferiority is defined as a 3-year survival rate that is no more than 15 percentage points below the expected 67% 3-year survival rate among patients in the standard surgery arm (data based on the CROSS-trial) [3, 4]. To allow for a 12% drop-out (e.g. patients in the active surveillance-arm who request immediate surgery in the absence of clinically proven or suspected regrowth) 300 patients are required for randomisation. Based on preliminary data from the current preSANO-trial, we expect that 50% of all included patients will have cCR during CRE-II, leading to a total required inclusion of 600 patients.

To reduce the number of newly included patients and to optimally use the data from the preSANO-trial, all recently (≥ May 2015) included patients with cCR during CRE-II from the current preSANO-trial who underwent bite-on-bite biopsies during CRE-I and CRE-II will be included in the control arm (*n* = 60 patients). Assuming a 50% cCR rate, the total number of required patients to be newly included in the SANO-trial will drop from 600 to 480 patients. Consequently, patients with cCR are randomised at an institutional level in a 3:5 ratio.

No interim analyses are planned for survival outcomes.

#### Data analysis

The difference in survival over a 3-year horizon between the control arm and the experimental treatment arm will be analysed with a mixed-effects Cox regression model. Use of a mixed regression model – including an institution-level random effect – is required to capture the potential between-institutional variation in survival [26]. To correct for potential selection bias, the treatment effect will be estimated with adjustment for prognostic factors for survival, i.e. age, sex, histologic subtype of tumour, clinical N stage, and WHO performance score. We will also use the mixed-effects Cox regression model to study potential differences in treatment effect between subgroups of patients. Subgroups are predefined according to age, sex, histologic subtype of tumour, clinical N stage, and WHO performance score. HRQOL data will be analysed according to the EuroQol, EORTC and Cancer Worry Scale scoring manuals [19–22]. Repeated measurement analysis will be used to evaluate within and between group differences. Data will be analysed following the intention-to-treat principle, including protocol deviators. A per protocol analysis will be performed as a secondary analysis.

### Ethical and regulatory considerations

The study has been approved by the medical ethics committee of the Erasmus MC (MEC2017–392) and has been registered in the Netherlands Trial Register (NTR 6803). The study will be conducted according to the principles of the Declaration of Helsinki (10th version, Fortaleza, 2013) and in accordance with the Dutch Medical Research Involving Human Subjects Act (WMO) and other applicable guidelines, regulations and Acts. In each participating centre, the local coordinating or principal investigator will be responsible for recruitment, data collection, follow-up of included patients, completion of case report forms and adherence to the study protocol. The supervising physician or any other physician of the multidisciplinary team will inform subjects about the study and ask for their consent using standard information letters and informed consent forms. Both patient information letters and informed consent forms are attached as separate documents.

An independent safety committee will be established to perform on-going safety surveillance and to perform interim analyses to assess the safety data and the stopping rules as described in “safety and stopping rules”. Each stopping rule will be repeatedly tested when the first 10, 20, 30 and 50 events for that particular stopping rule have occurred (i.e. [ad 1 and 2] detection of locoregional regrowth, [ad 3] the performance of delayed surgery or [ad 4] the detection of distant metastases).

The project leader (JL) is responsible for the study design and conduct of the trial, for the preparation of the protocol and revisions and for preparation of case report forms. Revisions of the study protocol will be communicated to all local chief investigators. The Clinical Trial Centre (CTC) of the Erasmus MC – University Medical Centre Rotterdam is responsible for the data master file, data verification and randomisation. Randomisation will be performed via a computer-generated random numbers sequence. Data will be collected using individual trial case numbers on standardised case report forms collated centrally by the CTC. Patients will not be individually identifiable. The final dataset will be available to all study investigators but will not be analysed per centre. Authorships will be defined following the International Committee of Medical Journal Editors guidelines [27]. Results will be communicated via international conferences, via publications and via the NTR.

## Discussion

Trials comparing surgical and non-surgical treatment modalities often fail due to low accrual if randomisation is at the patient level, which might be explained by patient preferences for an intervention [28–30]. Therefore, a stepped-wedge cluster design is applied in the present trial [31]. In a stepped-wedge design, randomisation takes place at the institutional level, and not at the patient level. Consequently, at the moment of inclusion patients know which treatment arm they will be assigned to, thereby overcoming uncertainty about which treatment patients will undergo. We expect that this will improve patients’ willingness to participate. When proven successful, the stepped-wedge design might be used as a new standard for comparing surgical with conservative treatments in clinical trials.

We will include both patients with SCC and patients with AC, since SCC and AC both respond to nCRT and no statistically significant differential effects were found in the CROSS-trial. Both patients with SCC and AC have a substantial pCR rate (49% and 23% in CROSS respectively) [3]. Moreover, preliminary results of the preSANO-trial suggests that residual disease can be diagnosed with comparable accuracy in patients with both histological subtypes.

Furthermore, in combination with the relatively low frequency of toxicity of the CROSS-regimen (91% completed the full nCRT-regimen), the high pCR-rate supports the use of the relatively low radiation dose of 41.4 Gy [3]. The beneficial effectivity/toxicity ratio is the rationale to apply the CROSS-regimen in the SANO-trial, and not a definitive chemoradiotherapy regimen (≥50 Gy of radiotherapy). The latter could increase the pCR-rate, but probably at the cost of a substantial increase in toxicity and postoperative complications, leading to a less beneficial effectivity/toxicity ratio. It should be noted that postponement of surgical resection, as will be performed in patients who develop locoregional regrowth in the absence of distant metastases, has been suggested to increase the incidence of postoperative complications. However, this phenomenon has been reported primarily after treatment with high-dose of definitive chemoradiotherapy (so called salvage esophagectomy) in low-volume centres [32, 33]. The SANO-trial will reveal whether this also applies to a lower dose of radiotherapy (CROSS regimen) in high-volume centres.

If the SANO-trial shows that active surveillance after nCRT for oesophageal cancer leads to non-inferior survival compared to standard oesophagectomy, this organ-sparing approach could be implemented as a standard of care. Of note, the French ESOSTRATE-trial is also comparing active surveillance with standard surgery in patients with cCR after nCRT. The ESOSTRATE-trial aims to include a total of 300 patients with SCC or AC with cCR after nCRT https://clinicaltrials.gov/ct2/show/NCT02551458. The primary endpoint is overall survival, as in the SANO-trial. Combining results from the ESOSTRATE-trial and the SANO-trial would lead to more certainty. Recently, we have shown that 54% and 61% of all patients are willing to trade-off 15% and 10% overall survival, respectively, to undergo active surveillance instead of standard surgery [34]. Therefore, the statistical power of the SANO-trial is for a non-inferiority margin of 15%; combination with the French ESOSTRATE-trial would reduce this margin to 10%. Hence, the future combination of results with the ESOSTRATE-trial is important to further increase our knowledge of an active surveillance approach beyond what we will learn from the SANO-trial only.

## Abbreviations

AC: AdenoCarcinoma
cCR: Clinically Complete Response
CRE: Clinical Response Evaluation
CROSS: ChemoRadiotherapy for Oesophageal cancer followed by Surgery Study [3]
DUCA: Dutch Upper-GI Cancer Audit
EUS: Endoscopic UltraSonography
FNA: Fine Needle Aspiration
Gy: Gray
MEC: Medical Ethics Committee
MTB: Multidisciplinary tumour board
nCRT: Neoadjuvant ChemoRadioTherapy
NTR: Netherlands Trial Register
OGD: OesophagoGastroDuodenoscopy
pCR: Pathologically Complete Response
PET-CT: Positron-Emission Tomography - Computed Tomography
SANO: Surgery As Needed approach in Oesophageal cancer patients
SCC: Squamous Cell Carcinoma
TNM: Tumour Node Metastasis classification system
TRG: Tumour Regression Grade
UICC: Union for International Cancer Control
WHO: World Health Organization
